# Methodological Considerations Regarding Cognitive Interventions in Dementia

**DOI:** 10.3389/fnagi.2014.00212

**Published:** 2014-08-13

**Authors:** Agustín Ibanez, Pablo Richly, María Roca, Facundo Manes

**Affiliations:** ^1^Laboratory of Experimental Psychology and Neuroscience (LPEN), Institute of Cognitive Neurology (INECO), Favaloro University, Buenos Aires, Argentina; ^2^UDP-INECO Foundation Core on Neuroscience (UIFCoN), Diego Portales University, Santiago, Chile; ^3^National Scientific and Technical Research Council (CONICET), Buenos Aires, Argentina; ^4^Universidad Autónoma del Caribe, Barranquilla, Colombia; ^5^Australian Research Council Centre of Excellence in Cognition and its Disorders, Sydney, NSW, Australia

**Keywords:** cognitive intervention, dementia, methods development, pre/post measurement, cognition, neuropsychology, neuroscience

Dementia causes massive cognitive, affective, and social impairment as well as concomitant functional decline. Cognitive interventions, together with pharmacological treatments, are acknowledged as important tools to delay mental weakening in dementing populations and to preserve the life quality of patients and their relatives (Prince et al., 2011; Woods et al., 2012). Given the socioeconomic impact of dementia on the health system, it is critical to assess cognitive intervention techniques in terms of cost-efficiency (Hurd et al., 2013). Nevertheless, most recent reports have neglected this issue, showing small, non-replicated, or even null results (Olazarán et al., 2010). Arguably, this is partly so because researchers are more specialized in the study of impairments than in the design of intervention programs. Consequently, there is no agreement on how to define cognitive intervention or how to measure its success (Giordano et al., 2010; Fernández-Prado et al., 2012).

A problem implicitly evident in relevant meta-analyses is that methodological decisions affect the results’ reliability and replication (Bahar-Fuchs et al., 2013a). Usually, an intervention program involves participant inclusion criteria, pre-intervention measurements, training sessions, post-intervention measurements, and pre–post statistical analyses to unravel the possible intervention effects. However, systematic methodological considerations for cognitive intervention in dementia are scant (Wollen, 2010). To increase the efficiency of cognitive stimulation assessment, this paper sets forth guidelines for cognitive intervention design, control group formation, control condition manipulation, pre/post-intervention measurement, and statistical analysis (see Table 1).

**Table 1:** **Relevant factors for improving research quality of cognitive interventions**.

Participants	Larger sample sizes allow for better control of confounding variables
	The control and the intervention groups must be matched for critical clinical variables (dementia diagnosis, disease severity, pharmacological treatment, caregiver support, etc.)
Methodological design	The assessment should be performed by specialists with specific training on the intervention
	The intervention should be as intense as possible and total duration should be long enough to produce changes in the assessed cognitive domains
	When interventions are focused on general aspects, a more controlled, multi-measured, and multivariate design with a large sample size should be considered
	Application manuals and standardized protocols are required in order to maximize training suitability and protocol comparability
Control group and intervention	Both groups must be comparable regarding critical clinical variables that can affect cognition
	Both groups should perform an activity that resembles the intervention in terms of intensity, duration, and social–physical environments
	Other confounding factors must be controlled for, such as adherence, incidental effects of external/personal events, and differences in domains other than the content of intervention
Pre- and post-measurements	Different evaluators should be considered to assess pre/post measures as well as cognitive intervention
	Measures targeting the specific cognitive domain trained should be used alongside more general measures
	Both self-reports from patients and caregivers and objective measures (ranging from neuropsychology and experimental design to brain function techniques, such as fMRI or EEG) must be considered
Statistical concerns	Statistical size effects are crucial in pre/post design
	Multiple comparisons, multivariate designs, and corrections for multiple comparisons should be performed only with larger samples
	For small samples, statistical assessment should be done at group-comparison level, with a focus on a single independent variable indexing the intervention assessment
	For small samples with a control group and pre/post assessment, analysis of multiple single cases should be a subsidiary strategy

## Participants

Participant selection is a critical issue, which affects study design. First, the sample size should be sufficiently large (approximately >100). This allows to control for possible confounders and to prevent the sample from becoming too small during follow-up. However, this is often not easy to achieve, given the difficulty to recruit patients with similar clinical characteristics (e.g., dementia subtype, pharmacological treatment and doses, disease duration and severity, and caregiver support). Current reports usually consider really modest samples (Niu et al., 2010; Dröes et al., 2011; Viola et al., 2011).

Different approaches should be considered for small and large groups. In small groups, intervention should be focused and specific (e.g., restricted to one cognitive domain) and based on relevant pre/post measures. For their own part, larger samples allow to track and control for confounding variables. Although a full clinical characterization is always desirable, the impact of these variables can be tracked only with large sample sizes.

Moreover, depending on the dementia subtype, diagnosis can be probable [e.g., behavioral variant of frontotemporal dementia (bvFTD)] or confirmed (e.g., Huntington’s disease with confirmed mutation). For probable diagnosis, more clinical/neuropsychological control measures should be considered. Moreover, intervention effects can be subtle and difficult to identify in conditions with rapid progression. Thus, different control and intervention strategies should be applied depending on diagnosis and sample size.

## Methodological Design

Intervention design is another critical aspect. The assessment should be done by specialists, with specific training on the intervention program, and the evaluation process should be monitored.

Regarding duration, the intervention should be as intense as possible (e.g., daily and with a repeated design) to produce stronger effects relative to the pre-intervention baseline (Kanaan et al., 2014). In addition, the intervention should be long enough to produce changes in the assessed cognitive domains, but total duration should be reduced for small groups, which have not been controlled for individual differences). The longer the intervention, the higher the probability that uncontrolled individual/personal events will affect the results (Luttenberger et al., 2012).

Another important factor is specificity versus generality. Interventions focused on general aspects (quality of life and general cognitive status) are more methodologically demanding, because of the complexities of general skill training and measurement (Carrion et al., 2013). Such situations call for a more controlled, multi-measured, and multivariate design (involving larger samples). Training in specific domains (e.g., working memory and emotional recognition) can be simpler for several reasons. There are relatively specific domains differentially affected in certain dementia subtypes [e.g., social cognition in bvFTD (Ibanez and Manes, 2012); memory in Alzheimer’s disease (Parra et al., 2009)]. In these cases, targeted interventions may allow for more direct and intense training. Moreover, participant monitoring is more straightforward, pre/post measures can be directly related with the content of the intervention, and these effects can be more easily tracked with specific instruments.

Thus, intervention should be assessed by trained and controlled professionals, capable of assessing the trade-off between intensity, duration, and specificity versus generality in program design. A related problem is the heterogeneity of the interventions and their poor descriptions. Application manuals/handbooks and standardized protocols are required to maximize training efficiency and comparability of protocols (Hunsley and Rumstein-McKean, 1999; Town et al., 2012).

## Control Group and Control Variables

The inclusion of a control group and control conditions increases the reliability and validity of the results. Repeated assessment and random aspects of personal experience have an impact on several cognitive domains. Thus, differences between pre- and post-measurements are not informative on their own. A control group should always be considered and, if possible, subjects must be randomly assigned to the experimental and control groups. The mock intervention or “placebo” effect usually emerges in studies involving unspecific social/recreational activities or no treatment at all (Bahar-Fuchs et al., 2013b). Control conditions should be carefully designed in order to hone specific intervention strategies.

Control group design should observe the following requisites: (a) patient characteristics should be measurable and comparable among groups; (b) the activities performed should be comparable with intervention tasks regarding intensity, duration, and social–physical environments; and (c) confounding factors should be controlled. Regarding the latter consideration, a gold standard approach should monitor groups regarding adherence, incidental effects of external/personal events, and differences in domains other than the one being assessed. The patients and relatives’ self-report scales and checklists prove very helpful for this strategy. If sample size allows, relevant individual differences should be measured and controlled by means of statistical comparisons. The patients’ progression history, recent significant life events, and everyday behavior (diet, exercise, social, and recreational activities) should be controlled by means of group comparisons or multivariate analyses.

Therefore, the detection of intervention-specific effects requires a strict strategy including a truly comparable control group, a monitoring process, and, when possible, the control and weighing of confounding variables.

## Pre/Post Measures

The difference between pre- and post-intervention measures is perhaps the most important aspect to consider. A pre-intervention measure (or baseline) allows controlling for individual differences, and the effect of pre–post repetition can be disentangled by including a control group (Papp et al., 2009). Nevertheless, the most important issue is how to quantify the specific domain being trained.

One important factor is to favor the similarity between the measurements and the cognitive domain being tested. This is easier to address when a specific domain is being trained (e.g., working memory) because the similarity between the training and its measurement is restricted to an isolated aspect, and can be done by direct measurement of the task performance. Nevertheless, when general domains are considered (e.g., social functioning), the distance between the intervention and an isolated measure becomes evident (Owen et al., 2010), requiring complex or multiple measures (e.g., clinical variables, cognitive screening, life quality assessment, functionality measures, and relatives’ reports). A more ambitious design should include specific and general training as well as specific and general pre/post-measurements. Note that a design as such requires a larger sample size, which is rarely observed in current reports.

Another important sub-aspect is the degree of objective measurement. Self-reports are adequate to assess patients’ and relatives’ subjective experiences of changes, but they lack objectivity and can be biased by different factors. Subjective reports should be accompanied by objective (implicit and explicit) measures, ranging from experimental designs to brain measures. Basic general cognitive assessments (e.g., MMSE and fluid intelligence) are usually considered in intervention programs, but their generality renders them insensitive to subtle changes.

Recent advances in translational cognitive neuroscience are promissory. Neuroimaging methods have demonstrated the sensitivity to training effects, suggesting their potential role as systematic measures of intervention success (Belleville et al., 2011). Electromagnetic techniques offer a simple, inexpensive approach to tap training effects (Vecchio et al., 2013; Yener and Basar, 2013). Moreover, recent breakthroughs in engineering have given rise to training-sensitive mathematical applications, such as the case of brain connectivity measures (with both neuroimaging and electromagnetic techniques) (Pievani et al., 2011).

In brief, an adequate integrated approach to assess cognitive intervention effects requires the use of multi-level measures (self-reports, neuropsychology, and neuroscience) and a balance between specificity and generality.

## Statistical Issues

Finally, statistical soundness is crucial to attain reliability, validity, and replicability. Although sample size and the assessment of confounding variables drive most of the considerations, there are important decisions, which can bias the results. First of all, statistical size effects are more imperative in pre/post design than in basic research. Thus, they should be always reported, because they offer direct information about the power of the intervention.

Multiple comparisons and multivariate designs should be considered only with larger samples; otherwise, the statistical validity of the results will prove highly questionable. Corrections for multiple comparisons and selection of complex statistical designs (e.g., structural equation models, discriminant analysis, or support vector machine) should also be restricted to larger samples. Multi-site studies represent a current solution to sample size problems.

For small samples, statistical assessment should be done at group-comparison level. The focus should be on a single independent variable indexing the intervention assessment. A valid alternative in the context of small samples with a control group and pre/post assessment would be the analysis of multiple single cases (McIntosh and Brooks, 2011; Sajjadi et al., 2013), allowing a deeper examination of intervention effects.

## Conclusion

Much more knowledge is required to understand the dramatic brain changes triggered by dementia and the parallel effects of training during cognitive intervention. The assessment of patients with dementia tends to underestimate several methodological considerations, sometimes yielding inconsistent, unreliable, or weak results. Here, we have highlighted important decisions, which may impact positively on research quality (see Table 1 for a summary of these issues). An enhanced control and systematization of these issues will improve our understanding of how cognitive training affects the patients’ and relatives’ quality of life.

## Conflict of Interest Statement

The authors declare that the research was conducted in the absence of any commercial or financial relationships that could be construed as a potential conflict of interest.

## References

[B1] Bahar-Fuchs A. Clare L. Woods B. (2013a). Cognitive training and cognitive rehabilitation for mild to moderate Alzheimer’s disease and vascular dementia. Cochrane Database Syst. Rev. 6, CD003260 10.1002/14651858.CD003260.pub2 23740535 PMC7144738

[B2] Bahar-Fuchs A. Clare L. Woods B. (2013b). Cognitive training and cognitive rehabilitation for persons with mild to moderate dementia of the Alzheimer’s or vascular type: a review. Alzheimers Res. Ther. 5, 35 10.1186/alzrt189 23924584 PMC3979126

[B3] Belleville S. Clément F. Mellah S. Gilbert B. Fontaine F. Gauthier S. (2011). Training-related brain plasticity in subjects at risk of developing Alzheimer’s disease. Brain 134(Pt 6), 1623–1634 10.1093/brain/awr037 21427462

[B4] Carrion C. Aymerich M. Baillés E. López-Bermejo A. (2013). Cognitive psychosocial intervention in dementia: a systematic review. Dement. Geriatr. Cogn. Disord. 36, 363–375 10.1159/000354365 24022505

[B5] Dröes R. M. van der Roest H. G. van Mierlo L. Meiland F. J. (2011). Memory problems in dementia: adaptation and coping strategies and psychosocial treatments. Expert Rev. Neurother. 11, 1769–1781 10.1586/ern.11.167 22091600

[B6] Fernández-Prado S. Conlon S. Mayán-Santos J. M. Gandoy-Crego M. (2012). The influence of a cognitive stimulation program on the quality of life perception among the elderly. Arch. Gerontol. Geriatr. 54, 181–184 10.1016/j.archger.2011.03.003 21458869

[B7] Giordano M. Dominguez L. J. Vitrano T. Curatolo M. Ferlisi A. Di Prima A. (2010). Combination of intensive cognitive rehabilitation and donepezil therapy in Alzheimer’s disease (AD). Arch. Gerontol. Geriatr. 51, 245–249 10.1016/j.archger.2009.11.008 19969381

[B8] Hunsley J. Rumstein-McKean O. (1999). Improving psychotherapeutic services via randomized clinical trials, treatment manuals, and component analysis designs. J. Clin. Psychol. 55, 1507–1517 10.1002/(SICI)1097-4679(199912)55:12<1507::AID-JCLP8>3.0.CO;2-A 10855484

[B9] Hurd M. D. Martorell P. Delavande A. Mullen K. J. Langa K. M. (2013). Monetary costs of dementia in the United States. N. Engl. J. Med. 368, 1326–1334 10.1056/NEJMsa1204629 23550670 PMC3959992

[B10] Ibanez A. Manes F. (2012). Contextual social cognition and the behavioral variant of frontotemporal dementia. Neurology 78, 1354–1362 10.1212/WNL.0b013e3182518375 22529204 PMC3335455

[B11] Kanaan S. F. McDowd J. M. Colgrove Y. Burns J. M. Gajewski B. Pohl P. S. (2014). Feasibility and efficacy of intensive cognitive training in early-stage Alzheimer’s disease. Am. J. Alzheimers Dis. Other Demen. 29, 150–158 10.1177/1533317513506775 24667905 PMC10852592

[B12] Luttenberger K. Hofner B. Graessel E. (2012). Are the effects of a non-drug multimodal activation therapy of dementia sustainable? Follow-up study 10 months after completion of a randomised controlled trial. BMC Neurol. 12:151 10.1186/1471-2377-12-151 23217188 PMC3527171

[B13] McIntosh R. D. Brooks J. L. (2011). Current tests and trends in single-case neuropsychology. Cortex 47, 1151–1159 10.1016/j.cortex.2011.08.005 21930266

[B14] Niu Y. X. Tan J. P. Guan J. Q. Zhang Z. Q. Wang L. N. (2010). Cognitive stimulation therapy in the treatment of neuropsychiatric symptoms in Alzheimer’s disease: a randomized controlled trial. Clin. Rehabil. 24, 1102–1111 10.1177/0269215510376004 20713437

[B15] Olazarán J. Reisberg B. Clare L. Cruz I. Peña-Casanova J. Del Ser T. (2010). Nonpharmacological therapies in Alzheimer’s disease: a systematic review of efficacy. Dement. Geriatr. Cogn. Disord. 30, 161–178 10.1159/000316119 20838046

[B16] Owen A. M. Hampshire A. Grahn J. A. Stenton R. Dajani S. Burns A. S. (2010). Putting brain training to the test. Nature 465, 775–778 10.1038/nature09042 20407435 PMC2884087

[B17] Papp K. V. Walsh S. J. Snyder P. J. (2009). Immediate and delayed effects of cognitive interventions in healthy elderly: a review of current literature and future directions. Alzheimers Dement. 5, 50–60 10.1016/j.jalz.2008.10.008 19118809

[B18] Parra M. A. Abrahams S. Fabi K. Logie R. Luzzi S. Della Sala S. (2009). Short-term memory binding deficits in Alzheimer’s disease. Brain 132(Pt 4), 1057–1066 10.1093/brain/awp036 19293236

[B19] Pievani M. de Haan W. Wu T. Seeley W. W. Frisoni G. B. (2011). Functional network disruption in the degenerative dementias. Lancet Neurol. 10, 829–843 10.1016/S1474-4422(11)70158-2 21778116 PMC3219874

[B20] Prince M. Bryce R. Ferr C. (2011). World Alzheimer Report 2011. London, UK: Alzheimer’s Disease International, 69

[B21] Sajjadi S. A. Acosta-Cabronero J. Patterson K. Diaz-de-Grenu L. Z. Williams G. B. Nestor P. J. (2013). Diffusion tensor magnetic resonance imaging for single subject diagnosis in neurodegenerative diseases. Brain 136(Pt 7), 2253–2261 10.1093/brain/awt118 23729473

[B22] Town J. M. Diener M. J. Abbass A. Leichsenring F. Driessen E. Rabung S. (2012). A meta-analysis of psychodynamic psychotherapy outcomes: evaluating the effects of research-specific procedures. Psychotherapy (Chic) 49, 276–290 10.1037/a0029564 22962969

[B23] Vecchio F. Babiloni C. Lizio R. Fallani F. V. Blinowska K. Verrienti G. (2013). Resting state cortical EEG rhythms in Alzheimer’s disease: toward EEG markers for clinical applications: a review. Suppl. Clin. Neurophysiol. 62, 223–236 10.1016/B978-0-7020-5307-8.00015-6 24053043

[B24] Viola L. F. Nunes P. V. Yassuda M. S. Aprahamian I. Santos F. S. Santos G. D. (2011). Effects of a multidisciplinary cognitive rehabilitation program for patients with mild Alzheimer’s disease. Clinics (Sao Paulo) 66, 1395–1400 10.1590/S1807-59322011000800015 21915490 PMC3161218

[B25] Wollen K. A. (2010). Alzheimer’s disease: the pros and cons of pharmaceutical, nutritional, botanical, and stimulatory therapies, with a discussion of treatment strategies from the perspective of patients and practitioners. Altern. Med. Rev. 15, 223–244 21155625

[B26] Woods B. Aguirre E. Spector A. E. Orrell M. (2012). Cognitive stimulation to improve cognitive functioning in people with dementia. Cochrane Database Syst. Rev. 2, CD005562 10.1002/14651858.CD005562.pub2 22336813

[B27] Yener G. G. Basar E. (2013). Biomarkers in Alzheimer’s disease with a special emphasis on event-related oscillatory responses. Suppl. Clin. Neurophysiol. 62, 237–273 10.1016/B978-0-7020-5307-8.00020-X 24053044

